# Surface Functionalized Polyhydroxyalkanoate Nanoparticles via SpyTag–SpyCatcher System for Targeted Breast Cancer Treatment

**DOI:** 10.3390/pharmaceutics17060721

**Published:** 2025-05-29

**Authors:** Jin Young Heo, Min Kyung Sung, Seonhye Jang, Hansol Kim, Youngdo Jeong, Dong-Jin Jang, Sang-Jae Lee, Seong-Bo Kim, Sung Tae Kim

**Affiliations:** 1 Department of Nanoscience and Engineering, Inje University, Gimhae 50834, Republic of Korea; camp.heo@outlook.com (J.Y.H.); mksung48@naver.com (M.K.S.); cooco00@naver.com (S.J.); hsk@inje.ac.kr (H.K.); 2 Department of Pharmaceutical Engineering, Inje University, Gimhae 50834, Republic of Korea; 3 Center for Advanced Biomolecular Recognition, Biomedical Research Division, Korea Institute of Science and Technology (KIST), Seoul 02792, Republic of Korea; zerodegree@kist.re.kr; 4 Department of Bio-Health Technology, College of Biomedical Science, Kangwon National University, Chunchoen 24341, Republic of Korea; djjang@kangwon.ac.kr; 5 Major in Food Biotechnology and Research Center for Extremophiles and Marine Microbiology, Silla University, Busan 46958, Republic of Korea; sans76@gmail.com; 6 Bio-Living Engineering, Global Leaders College, Yonsei University, Seoul 03722, Republic of Korea; 7 Department of Integrative Biotechnology, Yonsei University, Incheon 21983, Republic of Korea

**Keywords:** polyhydroxyalkanoate, HER2, Affibody, TAT, SpyTag–SpyCatcher

## Abstract

**Background/Objectives:** Biodegradable polymers have emerged as promising platforms for drug delivery. Produced by microbiomes, polyhydroxyalkanoates (PHAs) offer excellent biocompatibility, biodegradability, and environmental sustainability. In this study, we report the surface functionalization of PHA-based nanoparticles (NPs) using the SpyTag–SpyCatcher system to enhance cellular uptake. **Methods:** Initial conjugation with mEGFP-SpyTag enabled visualization, followed by decoration with HER2-specific Affibody-SpyCatcher and/or TAT-SpyCatcher peptides. The prepared NPs retained a diameter of <200 nm and a negatively charged surface. **Results:** Affibody-functionalized NPs significantly enhanced internalization and cytotoxicity in HER2-overexpressing SK-BR-3 cells, whereas TAT-functionalized NPs promoted uptake across various cell types, independently of HER2 expression. Dual-functionalized NPs exhibited synergistic or attenuated effects based on the HER2 expression levels, highlighting the critical role of ligand composition in targeted delivery. **Conclusions:** The results of this study demonstrate that the SpyTag–SpyCatcher-mediated surface engineering of PHA NPs offers a modular and robust strategy for active targeting in nanomedicine.

## 1. Introduction

Biodegradable polymers, a class of eco-friendly polymers that can be degraded into useful substances by microorganisms, are promising materials for drug delivery systems [1,2,3,4]. These polymers undergo chemical and/or enzymatic degradation in the environment, yielding biocompatible by-products [5]. They can also be categorized into natural polymers, such as polysaccharides (e.g., starch, cellulose, and chitosan), protein-based polymers (e.g., collagen and silk fibroin), and synthetic polymers, such as polylactic acid, poly(lactic-co-glycolic acid), and polyethylene glycol [6,7,8]. Their favorable biological and environmental properties make them high-demand materials, driving rapid progress in the development of novel biodegradable systems. These advances have intensified the interest in their application across biomedical and other interdisciplinary fields [9,10].

Drug delivery systems based on biodegradable polymers have undergone substantial advancement [11,12,13]. These systems are designed to encapsulate therapeutic agents, enabling their targeted delivery to specific cells, tissues, or organs [14,15]. Such strategies enhance drug stability and therapeutic efficacy through controlled release mechanisms and site-specific targeting [16,17]. The surface modification of polymeric carriers is key to active targeting [18]. Biofunctionalized polymeric nanoparticles (NPs), conjugated with targeting moieties, such as antibodies, have been widely studied for their ability to enhance cellular uptake and targeting precision [19,20,21,22]. The tunable physicochemical properties of polymeric surfaces allow precise and stable functionalization, thereby expanding their utility in biological systems [23]. Functional groups can be introduced via noncovalent or covalent strategies [24]. Covalent conjugation is generally preferred for anchoring active targeting ligands, including aptamers, affibodies, and antibodies, due to its greater stability and resistance to environmental fluctuations, such as pH and ionic strength [25]. These actively modified nanocarriers offer considerable potential for the precise delivery of therapeutic agents, enhancing the effectiveness of active targeting modalities.

Herein, we report on the development of surface-functionalized nanocarriers based on polyhydroxyalkanoate (PHA), a biodegradable polymer biosynthesized by microorganisms. To actively target and enhance cellular uptake in mammary cancer cells, the nanoparticle surface was functionalized with a human epidermal growth factor receptor 2 (HER2)-specific Affibody and/or the cell-penetrating peptide TAT using the SpyTag–SpyCatcher ligation system. The targeting efficiency of these bioorthogonal functionalized nanocarriers was assessed to evaluate their potential for use in the active targeting of breast cancer cells.

## 2. Materials and Methods

### 2.1. Materials

PHA (Poly[(R)-3-hydroxybutyric acid] (PHB) granules) was purchased from Goodfellow Cambridge Ltd. (Huntingdon, UK). Paclitaxel (PTX) was kindly supplied by Prof. Dong-Jin Jang at Kangwon National University, produced by Samyang Biopharmaceuticals (Seongnam, Republic of Korea). For functionalization, enhanced green fluorescent protein (mEGFP)-SpyTag, HER2 Affibody-SpyCatcher, and TAT-SpyCatcher were successfully synthesized (Table S1). Palmitic acid *N*-hydroxysuccinimide (PA-NHS) ester, used as a lipid linker, was purchased from Sigma-Aldrich (St. Louis, MO, USA), whereas sodium deoxycholate (NaDC), used for solubilization of the lipid linker, was from Tokyo Chemical Industry (TCI, Tokyo, Japan). Polyvinyl alcohol (PVA), which was used as a surfactant, and chloroform, which was used as the oil phase for solvent evaporation, were purchased from Duksan General Science (Duksan, Ansan, Republic of Korea). Pierce™ BCA Protein Assay Kits for protein quantification and HER2 antibody for extracellular HER2 expression analysis were obtained from Thermo Fisher Scientific (Waltham, MA, USA). Dulbecco’s modified Eagle’s medium (DMEM, Corning, NY, USA), penicillin–streptomycin (Corning Inc., Corning, NY, USA), fetal bovine serum (FBS, Corning, NY, USA), MEGM™ Mammary Epithelial Cell Growth Medium Bullet Kit™ (Lonza, Basel, Switzerland), Dulbecco’s phosphate-buffered saline (DPBS, Gibco, Waltham, MA, USA), and ultrapure water (Welgene Inc., Gyeongsan, Republic of Korea) were used for cell culturing. The Cell Counting Kit-8 (CCK-8) was obtained from DoGenBio (Seoul, Republic of Korea). All the reagents and chemicals used were of analytical grade.

### 2.2. Synthesis of SpyTag–Lipid Linker Conjugate

To prepare the SpyTag–lipid linker conjugate [26], mEGFP-SpyTag stock solution (3 mg equivalent) was thawed at room temperature and filtered using 10 kDa AMICON^®^ Ultra-0.5 Centrifugal Filters (Millipore, Burlington, MA, USA). The protein on the filter was collected and dissolved in 1 mL of 2% NaDC in 0.1× phosphate-buffered saline (PBS). The PA-NHS ester was added at a 5-fold molar excess relative to mEGFP-SpyTag into 2 mL of 2% NaDC in 0.1× PBS and sonicated until well mixed. The two solutions were mixed and incubated overnight at 37 °C with gentle agitation. The reaction mixture was then dialyzed against 0.15% NaDC in 1× PBS at 37 °C for 24 h to remove excess reactants and hydrolyzed ester. Post dialysis, the mEGFP-SpyTag–palmitic acid conjugate solution was filtered using 10 kDa AMICON^®^ Ultra-0.5 Centrifugal Filters and redispersed in 0.15% NaDC in 1× PBS. The final product was stored at 4 °C for further use.

### 2.3. Preparation and Characterization of SpyTag-Decorated PHA NPs

SpyTag-decorated PHA NPs were prepared using a two-step emulsion solvent evaporation method [27]. Briefly, PHA (10 mg) and PTX (0.1 mg) were dissolved in 2 mL of chloroform to form the oil phase. The oil phase was emulsified with 6 mL of an aqueous phase composed of 3 mL of 5% (*w*/*v*) PVA solution and 3 mL of mEGFP-SpyTag-palmitic acid conjugate solution (0.33 mg/mL). To prepare the control formulations, either an oil phase without PTX was used to generate blank NPs, or mEGFP-SpyTag solution was further used to generate unconjugated NPs.

The primary emulsion was sonicated at 300 watts for 1 min in an ice bath using a probe-type ultrasonic homogenizer (Scientz-11D; NingBo Scientz Biotechnology, Ningbo, Zhejiang, China). The resulting emulsion was poured into a 0.5% (*w*/*v*) PVA solution and stirred at 500 rpm for 3 h in a fume hood to evaporate the organic solvent and harden the particles. The resulting PHA NPs were collected by centrifugation at 24,000× *g* for 3 h at 4 °C (1580R, Labogene ApS, Lillerød, Denmark) and washed thoroughly with double-distilled water to remove residual PVA. The purified NPs were stored at 4 °C for further analysis. To verify the SpyTag-decoration of NPs via the lipid linker conjugation, the fluorescence intensity of mEGFP-SpyTag was analyzed using a NovoCyte Advanteon flow cytometer (Agilent Technologies, Santa Clara, CA, USA). The fluorescence of functionalized NPs was compared to that of unconjugated NPs, and the data were analyzed using NovoExpress software (version 1.6.2, Agilent Technologies).

### 2.4. Quantification of mEGFP-SpyTag on PHA NPs

To determine the appropriate amount of HER2 Affibody-SpyCatcher and TAT-SpyCatcher for subsequent functionalization, the amount of mEGFP-SpyTag decorated on the surface of the PHA NPs was quantified using a modified BCA assay [28]. Both standard solutions and mEGFP-SpyTag-functionalized PHA NPs were incubated with BCA working reagents at 37 °C for 4 h. Next, absorbance at 562 nm was measured using a microplate reader (Synergy™ HTX Multi-Mode Microplate Reader, BioTek, Winooski, VT, USA). The amount of mEGFP-SpyTag was expressed as micrograms per milligram of PHA, and all measurements were performed in triplicate (n = 3).

### 2.5. Surface Functionalization of PHA NPs via the SpyTag–SpyCatcher System

Based on the quantification results derived for mEGFP-SpyTag, the surface functionalization of PHA NPs was performed via HER2 Affibody-SpyCatcher or TAT-SpyCatcher at a 1:1 molar ratio with mEGFP-SpyTag anchored on the PHA NP surface [29]. Each mixture was incubated at room temperature for 30 min, with gentle shaking to facilitate binding. Free HER2 Affibody-SpyCatcher and TAT-SpyCatcher were eliminated using 100 kDa AMICON^®^ Ultra-4 Centrifugal Filters (Millipore, Burlington, MA, USA). The functionalized NPs were resuspended in double-distilled water and stored at 4 °C for further use.

### 2.6. Physicochemical Properties of PHA NPs

#### 2.6.1. Size and Zeta Potential Measurement of PHA NPs

The particle size and polydispersity index (PDI) of PHA NPs were measured via dynamic light scattering (DLS) using a Zetasizer (Nano-ZS90, Malvern Instruments, Malvern, Worcestershire, UK) at a fixed angle of 90° and a temperature of 25 °C. Zeta potential values were determined at 25 °C using the same instrument. All samples were dispersed in double-distilled water prior to measurements. Each measurement was conducted in triplicate (n = 3), and the results are presented as mean ± standard deviation.

#### 2.6.2. Scanning Electron Microscopic Analysis of the PHA NPs

The morphological characteristics of the PHA NPs were examined using field-emission scanning electron microscopy (FE-SEM; S-4300SE, Hitachi, Tokyo, Japan). The samples were dried in a vacuum oven at 50 °C and sputter-coated with platinum–palladium (Pt/Pd) alloy prior to imaging.

#### 2.6.3. Storage Stability of PHA NPs

The storage stability of various types of PHA NPs was evaluated for 1 week at 4 °C. The particle size, PDI, and zeta potential were measured using a Zetasizer as described above. All samples were dispersed in double-distilled water and measured at 25 °C in triplicate (n = 3). The results are presented as mean ± standard deviation.

### 2.7. Determination of PTX Content in NPs

The encapsulation efficiency of PTX was quantitatively determined using high-performance liquid chromatography (HPLC, Agilent 1100 series, Agilent Technologies) [30]. Briefly, the purified NP suspension was centrifuged at 24,000× *g* for 3 h. The resulting PHA NP pellets were dissolved in dichloromethane (DCM), followed by the complete evaporation of the solvent under a nitrogen stream. The dried residue was resuspended in methanol and sonicated for 1 h using an ultrasonic water bath (Bransonic^®^ CPXH 3800, Branson Ultrasonics, Brookfield, CT, USA) to extract PTX. Following sonication, the samples were centrifuged at 24,000× *g* for 1 h, and the supernatant was filtered and used for HPLC analysis.

HPLC analysis was performed using a C_18_ column (Supersil^®^ 120 ODS II, 4.6 mm × 150 mm, 5 μm, LB Science, Seoul, Republic of Korea) maintained at 40 °C. The mobile phase consisted of a mixture of acetonitrile and water (50:50, *v*/*v*) with a flow rate of 1.0 mL/min. Each sample (20 μL) was injected and monitored at a detection wavelength of 227 nm using a UV detector. The HPLC system was controlled using Agilent ChemStation software (Rev. B.04.03). All measurements were performed in triplicate (n = 3). The encapsulation efficiency was calculated as the percentage of PTX encapsulated within the NPs relative to the initial amount of PTX used.

### 2.8. Serum Stability of PHA NPs

To assess the stability of PHA NPs under physiological conditions prior to the in vitro experiments, a serum stability study was performed [31]. PHA NPs were suspended in PBS containing various concentrations of fetal bovine serum (FBS) (0% and 10%) and incubated at 37 °C. Particle sizes were measured at designated time points (0, 60, 120, 240 and 480 min) using dynamic light scattering (DLS) with a Zetasizer. All measurements were conducted in triplicate (n = 3). The results are presented as mean ± standard deviation.

### 2.9. In Vitro Cell Culture

Breast cancer cell lines (SK-BR-3, MCF-7, and MDA-MB-231) and a noncancerous mammary epithelial cell line (MCF-10A) were used for in vitro experiments. SK-BR-3 and MCF-7 cells were obtained from Korean Cell Line Bank (Seoul, Republic of Korea). MCF-10A cells were obtained from the American Type Culture Collection (ATCC, Manassas, VA, USA), and MDA-MB-231 cells were kindly provided by Prof. H. Kim. All cell lines, except for MCF-10A, were cultured in DMEM supplemented with 10% FBS and 1% penicillin-streptomycin. MCF-10A cells were maintained in Mammary Epithelial Cell Growth Medium BulletKit (Lonza). All the cell cultures were incubated at 37 °C in a humidified atmosphere containing 5% CO_2_ (BB15; Thermo Fisher Scientific).

### 2.10. Cellular Uptake of Surface Functionalized PHA NPs

To evaluate the cellular uptake of PHA NPs, Nile Red-loaded formulations were prepared by incorporating 2 µg of Nile Red per 1 mg of PHA during NP fabrication [32], as described above. Unfunctionalized and surface-functionalized PHA NPs were analyzed using flow cytometry and confocal laser scanning microscopy (CLSM). For imaging [33,34], all cell lines were seeded on poly-*_L_*-lysine-coated cover glasses (PLL solution, Sigma-Aldrich) placed in 6-well plates and preincubated at 37 °C for 24 h. Cells were treated with unfunctionalized or functionalized NPs at a PTX concentration of 20 nM (SK-BR-3, MCF-10A, and MDA-MB-231) or 10 nM (MCF-7) for 4 h. After 4 h of incubation, LysoTracker^TM^ (Yellow HCK-123, Invitrogen, Waltham, MA, USA) was added at a final concentration of 100 nM and incubated for an additional 2 h to visualize lysosomes. The cover glasses were washed twice with PBS and stained with 4′,6-diamidino-2-phenylindole (DAPI, Fluoroshield™ with DAPI, Sigma-Aldrich) for 30 min at 25 °C. Confocal images were acquired using a 63× oil immersion objective on a CLSM system (LSM800, Zeiss, Jena, Germany) under the following detection settings: DAPI, 405 nm laser; excitation, 353 nm; emission, 465 nm; detection, 400–500 nm. Nile Red: 559 nm laser; excitation 559 nm, emission 636 nm; detection 565–700 nm. LysoTracker^TM^: 488 nm laser; excitation 488 nm, emission 565 nm; detection 600–700 nm.

For quantitative analysis [35,36], all cell lines were seeded into 6-well plates and preincubated at 37 °C for 24 h. Cells were treated with NPs under the same PTX concentrations and conditions as described above. After 4 h of incubation, they were washed three times with PBS, and dissociated using 0.05% trypsin in 0.53 mM EDTA (Corning). The trypsinized cells were centrifuged and fixed with 4% paraformaldehyde (Thermo Fisher Scientific) in FACS buffer supplemented with 5% FBS and 0.1% sodium azide for 15 min at 25 °C. The fixed cells were washed and resuspended in 500 µL of cold DPBS. Cellular uptake was assessed using a flow cytometer, and the fluorescence intensity of Nile Red was detected using a phycoerythrin (PE) filter. A total of 10,000 events per sample were analyzed, and the relative fluorescence intensities of the unfunctionalized and functionalized NPs were compared using the NovoExpress^®^ software (version 1.6.2, Agilent Technologies).

### 2.11. Cell Cytotoxicity Assay

The cytotoxicity of unfunctionalized and surface-functionalized PHA NPs was evaluated using a Cell Counting Kit-8 (CCK-8) assay [37,38,39]. SK-BR-3 and MCF-10A cells (6 × 10^3^ cells/well), MDA-MB-231 cells (4 × 10^3^ cells/well), and MCF-7 cells (2 × 10^3^ cells/well) were seeded in 96-well plates and preincubated at 37 °C for 24 h. Following preincubation, the cells were treated with either unfunctionalized or surface-functionalized NPs containing PTX at a concentration of 20 nM for MCF-10A, SK-BR-3, and MDA-MB-231 cells, and 10 nM for MCF-7 cells. PHA NPs were incubated with the cells for 4 h, followed by removing the treatment medium and replacing it with fresh culture medium. The cells were then incubated for 72 h at 37 °C. Following incubation, CCK-8 reagent was added to each well at a ratio of 1:10 (CCK-8 reagent:medium, *v*/*v*) and incubated at 37 °C. Absorbance was measured at 450 nm using a multimode microplate reader. Cell viability was calculated as the percentage of the absorbance of treated cells relative to that of untreated control cells.

### 2.12. Statistical Analysis

Student’s *t*-test was used to compare two groups, employing a one-tailed unpaired test. Differences between the groups were considered statistically significant at * *p* < 0.05, ** *p* < 0.01, and *** *p* < 0.001.

## 3. Results

### 3.1. Surface Immobilization Using Spy Tag–Lipid Linker Conjugation

Prior to the immobilization of mEGFP-SpyTag onto the surface of the NPs via lipid linker conjugation, four formulations were prepared: PHA NPs, NPs with PTX, mEGFP NPs with PTX (unconjugated NP), and mEGFP-PA NPs with PTX (lipid linker conjugated NP). To confirm this immobilization, fluorescence intensity on the NP surfaces was analyzed using flow cytometry (Figure S1). The flow cytometry results show that all formulations exhibited comparable side scatter (SSC) intensities, suggesting similar particle sizes across groups. Notably, FITC fluorescence was observed exclusively in mEGFP-PA NP with PTX, indicating that the lipid linker strategy successfully enabled the surface decoration of mEGFP-SpyTag on the NPs. Furthermore, protein quantification confirmed that 34.82 ± 0.29 µg of mEGFP-SpyTag was immobilized per 1 mg of NPs.

### 3.2. Physicochemical Properties of NPs

Based on these decorated NPs, further functionalization was conducted using HER2 Affibody-SpyCatcher, TAT-SpyCatcher, or a 1:1 mixture of the two ligands. The physicochemical characteristics of each formulation were analyzed using DLS and zeta potential measurements (Table 1). PTX encapsulation resulted in a subtle increase in hydrodynamic diameter. Blank NPs (F1) had an average size of 120.07 ± 0.25 nm, whereas PTX-loaded NPs (F2) reached 134.60 ± 1.67 nm, although the presence of PTX had minimal effect on zeta potential and PDI. Decoration of the NP surface with mEGFP-SpyTag led to a slight increase in size (142.60 ± 1.87 nm) and a reduction in zeta potential values from −17.13 ± 1.63 mV to −11.77 ± 0.42 mV, presumably due to the exposure of protein domains on the particle surface. Although the PDI slightly increased (0.15 ± 0.02), it remained within the monodisperse range [40]. As shown in Figure S2, the FE-SEM images further confirm that the PHA NPs exhibited a uniform spherical morphology.

Surface functionalization with HER2 Affibody-SpyCatcher (F4), TAT-SpyCatcher (F5), or both ligands (F6) yielded no significant differences in the particle size and PDI value, and did not significantly alter the particle size compared to mEGFP-SpyTag-decorated NPs; however, the zeta potential values showed distinct shifts based on the type of ligand. HER2 Affibody-functionalized NPs (F4) exhibited a more negative surface charge (−14.77 ± 0.93 mV), whereas TAT-functionalized NPs (F5) displayed a less negative potential (−7.47 ± 0.77 mV), consistent with the cationic nature of TAT [41,42]. Dual-functionalized NPs (F6) showed an intermediate zeta potential of −11.60 ± 0.62 mV, reflecting a balanced contribution of both ligands. These results indicate that the ligand-dependent modulation of the surface charge can be achieved without significantly affecting particle size or colloidal stability. Moreover, successful conjugation was confirmed using SDS-PAGE, validating the SpyTag–SpyCatcher interaction between mEGFP-SpyTag and each SpyCatcher ligand (Figure S3). These results demonstrate the achievement of surface decoration with GFP-SpyTag and subsequent functionalization with the HER2 Affibody and/or TAT.

### 3.3. Physicochemical Stability of NPs

**Figure 1:**
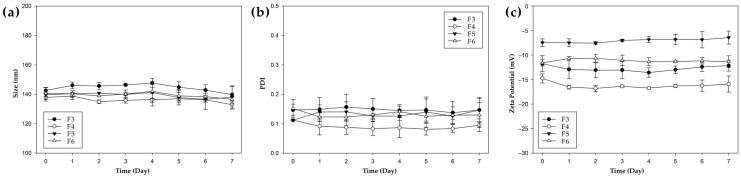
Storage stability of PHA NPs at 4 °C over 7 days; (**a**) particle size, (**b**) PDI values, (**c**) zeta potential value.

To assess the storage stability of NPs, the physicochemical properties of unfunctionalized and functionalized formulations (F3–F6) were monitored over 7 days at 4 °C (Figure 1). Throughout the storage period, no significant changes were observed in particle size, PDI, or zeta potential across all formulations. The particle sizes remained within a narrow range (approximately 130–160 nm) with minimal fluctuations, and all PDI values were consistently maintained below 0.2, indicating stable monodispersity. These results demonstrate that all NP formulations maintained excellent colloidal stability under refrigerated conditions for at least 7 days.

### 3.4. Serum Stability of PHA NPs

Prior to in vitro cell experiments, the physical stability of PHA NPs was assessed under serum-containing conditions (Figure S4). When suspended in PBS supplemented with 0% or 10% FBS and incubated at 37 °C, no significant changes in particle size were observed over an 8 h period, indicating that the formulations maintained stable homogeneous dispersion characteristics.

### 3.5. In Vitro Cellular Uptake

Prior to evaluating the cellular uptake, HER2 expression levels across various cell lines were assessed using flow cytometry (Figure S5). To confirm this, various human breast cancer cell lines (SK-BR-3, MCF-7, and MDA-MB-231) and non-cancerous breast epithelial cells (MCF-10A) were treated with FITC-conjugated anti-HER2 monoclonal antibodies, and the fluorescence intensities were compared with those of untreated controls. SK-BR-3 cells exhibited significantly higher HER2 expression than other cell lines, whereas MCF-7 cells showed moderate expression. MDA-MB-231 and MCF-10A cells showed minimal HER2 expression. Based on these results, cellular uptake studies were conducted by treating each cell line with unfunctionalized PHA NPs (F3) or functionalized PHA NPs (F4–F6).

**Figure 2:**
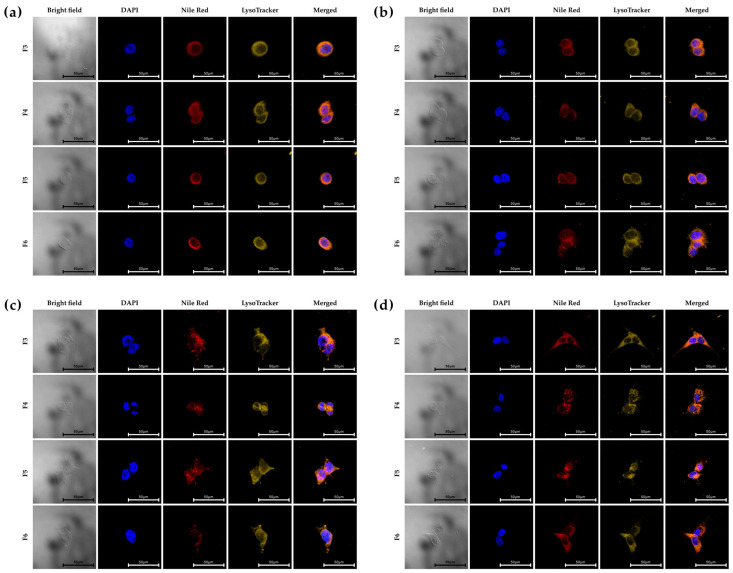
Cellular uptake of F3, F4, F5 and F6 in (**a**) SK-BR-3, (**b**) MCF-7, (**c**) MDA-MB-231, and (**d**) MCF-10A using CLSM. For tracking NileRed/PTX-loaded PHA NPs (red), the following dyes were used: DAPI (blue) for nuclei, LysoTracker^TM^ (yellow) for late endosomes/early lysosomes. Scale bar = 50 μm.

Confocal laser scanning microscopy was used to visualize the intracellular uptake following incubation with Nile Red-loaded NPs (F3 and F4–F6) (Figure 2). LysoTracker^TM^ staining confirmed whether NPs are localized within lysosomal compartments. Nile Red signals (red) were detected in the cytoplasm but not in the nuclei (stained with DAPI, blue). Most of the Nile Red signals co-localized with lysosomes (yellow), indicating successful endocytosis, although there were variations in surface ligands and zeta potential values among the NPs. These data demonstrate that NPs are effectively internalized across all cell lines.

**Figure 3:**
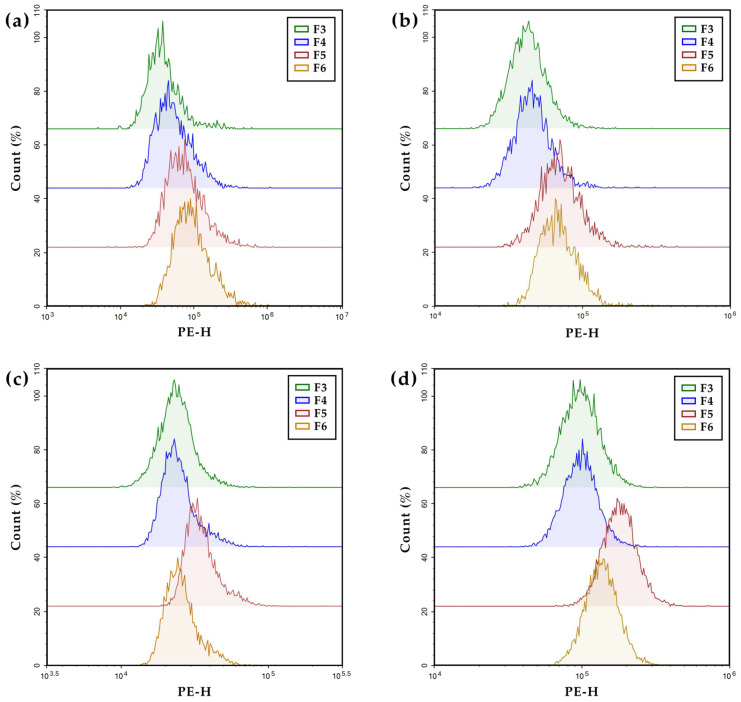
Intracellular uptake of F3, F4, F5 and F6 in (**a**) SK-BR-3, (**b**) MCF-7, (**c**) MDA-MB-231, and (**d**) MCF-10A cells using a flow cytometer.

**Figure 4:**
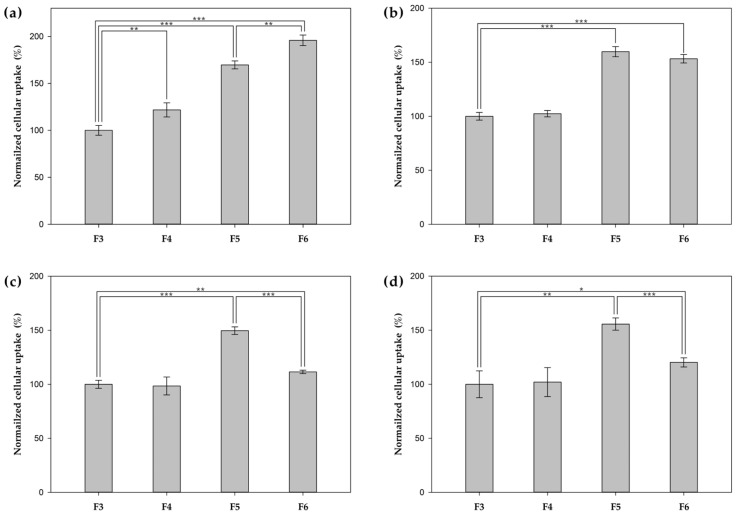
Normalized cellular uptake of F3, F4, F5 and F6 in (**a**) SK-BR-3, (**b**) MCF-7, (**c**) MDA-MB-231, and (**d**) MCF-10A, using a flow cytometer (* *p* < 0.05, ** *p* < 0.01, and *** *p* < 0.001).

To evaluate the relative cellular uptake of PHA NPs, flow cytometry was performed on both surface-functionalized (F4–F6) and unfunctionalized (F3) formulations across four cell lines (Figure 3 and Figure 4). To assess HER2-targeting efficiency, the uptake of HER2 Affibody-functionalized NPs (F4, Affibody^+^) was compared with that of unfunctionalized NPs (F3, Affibody^−^). In HER2-overexpressing SK-BR-3 cells, F4 treatment resulted in a 22% increase in uptake compared to F3. In contrast, negligible differences were observed in MCF-7, MDA-MB-231, and MCF-10A cells, which express low to intermediate levels of HER2. To examine the cell-penetrating ability of TAT-functionalized NPs (F5, TAT^+^), their uptake was compared with that of unfunctionalized NPs (F3, TAT^−^) across all tested cell lines. F5 treatment markedly enhanced cellular internalization in every cell line, with fluorescence intensities increasing by approximately 70% in SK-BR-3, 60% in MCF-7, 50% in MDA-MB-231, and 56% in MCF-10A relative to F3. A comparison between TAT-only NPs (F5) and dual-functionalized NPs (F6, Affibody^+^/TAT^+^) revealed HER2 expression-dependent trends. In SK-BR-3 cells, F6 induced greater uptake than F5 (196% vs. 170%), suggesting additive effects of dual ligands. In MCF-7 cells, which exhibit moderate HER2 expression, uptake levels between F6 and F5 were comparable (153% vs. 159%). However, in low HER2-expressing cell lines (MDA-MB-231 and MCF-10A), F6 treatment showed reduced uptake when compared to that of F5 (MDA-MB-231, 112% vs. 150%; MCF-10A, 120% vs. 156%), presumably due to a decreased surface density of TAT ligands following dual functionalization.

### 3.6. In Vitro Cytotoxicity Study

**Figure 5:**
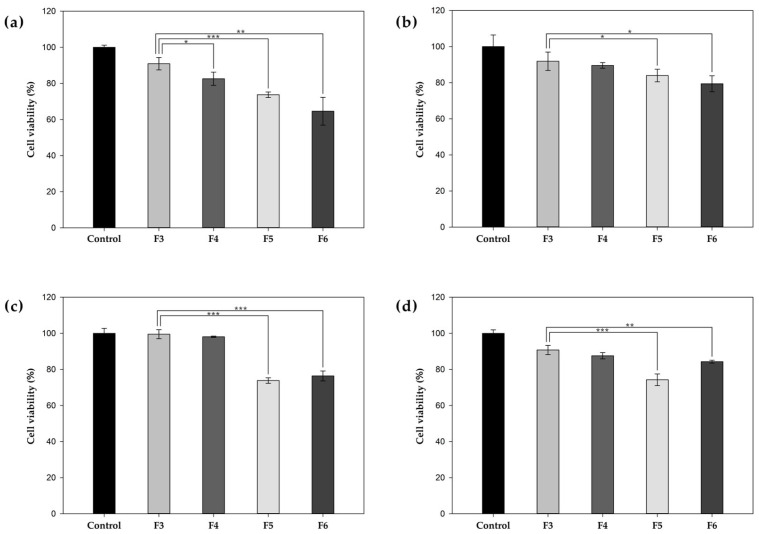
The cytotoxicity of F3, F4, F5 and F6 was evaluated in (**a**) SK-BR-3, (**b**) MCF-7, (**c**) MDA-MB-231, and (**d**) MCF-10A cells after treatment for 4 h. Cell viability was assessed using the Cell Counting Kit-8 (CCK-8) assay, and the results were analyzed to determine the cytotoxic effects of the treatments (* *p* < 0.05, ** *p* < 0.01, and *** *p* < 0.001).

To assess the PTX delivery efficacy of PHA NPs, both surface-functionalized (F4–F6) and unfunctionalized (F3) NPs were administered at equivalent PTX concentrations to multiple cell lines, and their cytotoxic effects were evaluated (Figure 5). To validate active targeting efficacy, the cytotoxicity of Affibody-functionalized NPs (F4, Affibody^+^) was compared with that of unfunctionalized NPs (F3, Affibody^−^). In SK-BR-3 cells, F4 treatment elicited markedly greater cytotoxicity than F3 treatment, with cell viabilities of approximately 9% and 17%, respectively. In contrast, no significant enhancement in cytotoxicity was observed in the MCF-7, MDA-MB-231, and MCF-10A cells. These findings indicate that HER2 Affibody functionalization substantially enhanced cellular uptake and cytotoxic effects in HER2-overexpressed cells. Similarly, the cytotoxicity of TAT-functionalized NPs (F5, TAT^+^) was compared with that of unfunctionalized NPs (F3, TAT^−^) across all cell lines. Following F3 treatment, the cell viability was approximately 9% in SK-BR-3, 8% in MCF-7, 1% in MDA-MB-231, and 9% in MCF-10A cells. In contrast, F5 treatment markedly increased cytotoxicity in all the tested cell lines, reducing cell viability to approximately 26% in SK-BR-3, 16% in MCF-7, 26% in MDA-MB-231, and 26% in MCF-10A cells. These results underscore the potent cell-penetrating capability of TAT-functionalized NPs. The cytotoxicity profiles of TAT-functionalized NPs (F5, TAT^+^) and dual-functionalized NPs (F6, Affibody^+^/TAT^+^) were also compared. In HER2-overexpressing SK-BR-3 cells and moderately expressing MCF-7 cells, dual-functionalized NPs (F6) induced greater cytotoxicity than TAT-functionalized NPs (F5), as reflected by reduced cell viabilities (SK-BR-3, 35% vs. 26%; MCF-7, 21% vs. 16%), likely due to the additional HER2-targeting capability provided by Affibody. Conversely, in low-HER2-expressing cell lines such as MDA-MB-231 and MCF-10A, F6 treatment resulted in diminished cytotoxic effects compared to F5 (MDA-MB-231, 24% vs. 26%; MCF-10A, 16% vs. 25%), presumably attributable to a lower surface density of TAT ligands following dual functionalization.

## 4. Discussion

The surface functionalization of NPs is a critical strategy for improving drug delivery efficiency and enabling targeted therapy. However, to the best of our knowledge, there have been no reports on the bioorthogonal conjugation of targeting peptides or proteins to the surface of PHA NPs, or on the simultaneous tracking of fluorescence. In this study, we utilized the SpyTag–SpyCatcher system to generate bioorthogonal nanoparticles for surface functionalization [37,43,44]. By incorporating mEGFP as a fluorescent molecule, we facilitated the tracking of intracellular delivery, such as in bioimaging. Moreover, the combination of the HER2-targeting Affibody and TAT peptides significantly enhanced the cellular uptake; therefore, this study represents the first investigation into the intracellular delivery of PHA-based NPs with these functionalities.

To prove this, cell experiments were conducted to assess the effects of surface-functionalized PHA NPs on cell-specific uptake and cytotoxicity. In SK-BR-3 cells, which have high HER2 expression [44,45], HER2 Affibody-functionalized NPs (F4) exhibited significantly increased cellular internalization and cytotoxicity compared to non-functionalized NPs (F3). These findings demonstrate that active targeting via the HER2 receptor was achieved [46,47]. In contrast, no prominent targeting effect was observed in mammary cell lines, such as MCF-10A and MDA-MB-231, well-known cells with lower HER2 expression [48,49]. NPs functionalized with cell-penetrating peptides such as TAT (F5) showed enhanced cellular uptake and cytotoxicity across all tested cell lines, regardless of HER2 expression levels. This result could be consistent with the known nonspecific and potent membrane-translocating ability of TAT, suggesting that TAT-functionalized NPs can be efficiently delivered into various cell types [50,51,52]. NPs co-functionalized with HER2 Affibody and TAT (F6) exhibited distinct behaviors depending on cellular HER2 expression levels. In HER2-overexpressing SK-BR-3 cells, F6 treatment significantly enhanced cellular uptake compared to the TAT-only-functionalized NPs (F5), presumably due to the synergistic effects of both ligands [53]. In contrast, no significant differences were observed in the MCF-7 cells with moderate HER2 expression. Conversely, in low-HER2-expressing cell lines (MCF-10A and MDA-MB-231), F6 demonstrated reduced internalization and cytotoxicity relative to F5, which could be attributed to the decreased surface density of TAT upon dual functionalization [54]. These findings underscore the critical role of the combination and density of surface ligands in optimizing the delivery efficiency based on cellular characteristics. Taken together, our study represents the first application of the bioorthogonal SpyTag–SpyCatcher system for the surface functionalization of biodegradable PHA-based NPs, offering a promising approach to enhancing drug delivery. This innovative strategy could serve as a versatile platform for developing polymer-based drug delivery systems, paving the way for more precise and effective therapeutic interventions.

## 5. Conclusions

In conclusion, we report the first implementation of the SpyTag–SpyCatcher system for the modular and stable surface functionalization of biodegradable PHA NPs with HER2 Affibody and/or TAT peptides. These surface-engineered NPs exhibited substantially enhanced cellular uptake and concomitant cytotoxicity, particularly in HER2-overexpressed breast cancer cells. Importantly, dual functionalization with HER2 Affibody and TAT imparted complementary functionalities, improving the delivery efficiency in a cell-type-dependent manner. Our findings establish that PHA-based nanocarriers integrated with robust and adaptable surface engineering strategies are versatile and promising platforms for next-generation targeted drug delivery and precision nanomedicine.

## Figures and Tables

**Table 1:** Physicochemical properties of PHA NPs.

PHA NPs		Size (nm)	Polydiversity Index (PDI)	Zeta Potential Value (ζ, mV)
F1	Blank NP	120.07 ± 0.25	0.06 ± 0.01	−16.37 ± 0.59
F2	NP with PTX	134.60 ± 1.67	0.07 ± 0.03	−17.13 ± 1.63
F3	GFP-SpyTag decorated NP with PTX	142.60 ± 1.87	0.15 ± 0.02	−11.77 ± 0.42
F4	HER2 Affibody functionalized * NP with PTX	137.90 ± 2.51	0.11 ± 0.01	−14.77 ± 0.93
F5	TAT functionalized * NP with PTX	139.67 ± 1.39	0.11 ± 0.01	−7.47 ± 0.77
F6	HER2 Affibody & TAT ** functionalized * NP with PTX	140.53 ± 4.13	0.15 ± 0.03	−11.60 ± 0.62

* SpyTag–SpyCatcher-based functionalization. ** both HER2 Affibody and TAT functionalization.

## Data Availability

The original contributions presented in this study are included in the article/Supplementary Material. Further inquiries can be directed to the corresponding author.

## Supplementary Materials

The following supporting information can be downloaded at: https://www.mdpi.com/article/10.3390/pharmaceutics17060721/s1, Table S1. Amino acid sequences of mEGFP-SpyTag, HER2 Affibody-SpyCatcher, and TAT-SpyCatcher; Figure S1. Fluorescent protein binding analysis on the surfaces of various types of PHA NPs by flow cytometry: (a) SSC-H histogram and (b) FITC-H histogram; Figure S2. FE-SEM images of NPs: (a) F2, (b) F3, (c) F4. Scale bars represent 500 nm; Figure S3. Sodium dodecyl-sulfate polyacrylamide gel electrophoresis (SDS-PAGE, 15%) to confirm the conjugation of mEGFP-SpyTag with TAT-SpyCatcher and HER2 Affibody-SpyCatcher via the SpyTag–SpyCatcher system; Figure S4. Serum stability of PHA NPs depending on time and serum concentration: (a) NP3, (b) NP4, (c) NP5 and (d) NP6; Figure S5. Flow cytometry analysis of HER2 expression levels in various cell lines: (a–d) FITC-H histograms of (a) SK-BR-3, (b) MCF-7, (c) MDA-MB-231, (d) MCF-10A cells and (e) bar graph of HER2 expression levels in each cell line using FITC-conjugated anti-HER2 monoclonal antibodies.
