# Supplementary material for: Surface Functionalized Polyhydroxyalkanoate Nanoparticles via SpyTag–SpyCatcher System for Targeted Breast Cancer Treatment
Source: Pharmaceutics. 2025 May 29;17(6):721. doi: 10.3390/pharmaceutics17060721 (PMC12196195; doi:10.3390/pharmaceutics17060721)
Supplement: Supplementary file 1 [file pharmaceutics-17-00721-s001.zip › pharmaceutics-3648944-supplementary.pdf]

Table S1. Amino acid sequences of mEGFP-SpyTag, HER2 Affibody-SpyCatcher, and TAT-SpyCatcher

Constructs	Amino acid sequence
mEGFP-SpyTag	FCLTLRRRYTMGSSHHHHHHSSMVSKGEELFTGVVPILVELDGDVNGHKFSVSGEGEGDATY GKLTLKFICTTGKLPVPWPTLVTTLTYGVQCFSRYPDHMKQHDFFKSAMPEGYVQERTIFFKD DGNYKTRAEVKFEGDTLVNRIELKGIDFKEDGNILGHKLEYNYNSHNVYIMADKQKNGIKVN FKIRHNIEDGSVQLADHYQQNTPIGDGPVLLPDNHYLSTQSKLSKDPNEKRDHMVLLEFVTA AGITLGMDELYKGGSAHIVMVDAYKPTK-EFELRRQACGRTRAPPPPPLRSGC-QSPK
HER2 Affibody-SpyCatcher	MGSSHHHHHHSQDPMVDTLSGLSSEQGQSGDMTIEEDSATHIKFSKRDEDGKELAGATMELR DSSGKTISTWISDGQVKDFYLYPGKYTFVETAAPDGYEVATAITFTVNEQGQVTVNGKATKGD AHIKNSTSTGGGSGGGASVDNKFNKEMRNAYWEIALLPNLNNQQKRAFIRSLYDDPSQSANL LAEAKKLNDAQAPKVD
TAT-SpyCatcher	YDSL-GNCERITIPL-K-FCLTLRRRYTMGSSHHHHHHGSGRKKRRQRRPPQGGS MVDTLSGLSSEQGQSGDMTIEEDSATHIKFSKRDEDGKELAGATMELRDSSGKTISTWISDGQV KDFYLYPGKYTFVETAAPDGYEVATAITFTVNEQGQVTVNGKATKGDAHI-VDKLAAA-CL

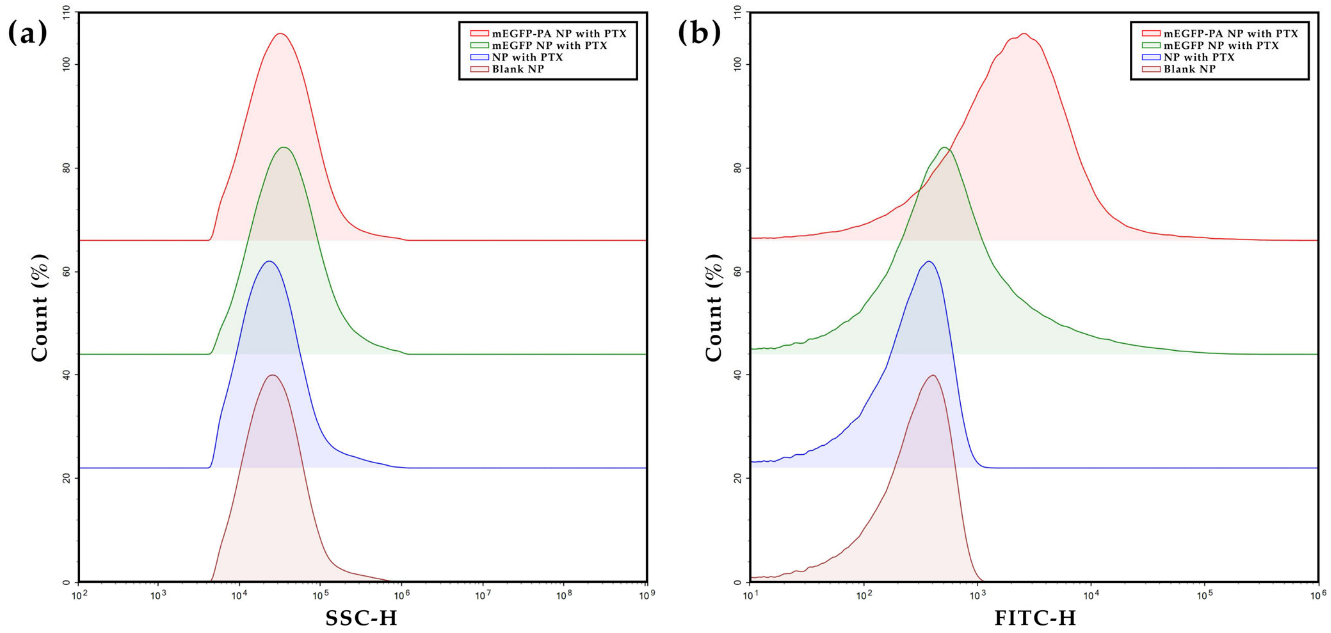

Figure S1. Fluorescent protein binding analysis on the surface of various types of PHA NPs by flow cytometry: (**a**) SSC-H histogram and (**b**) FITC-H histogram

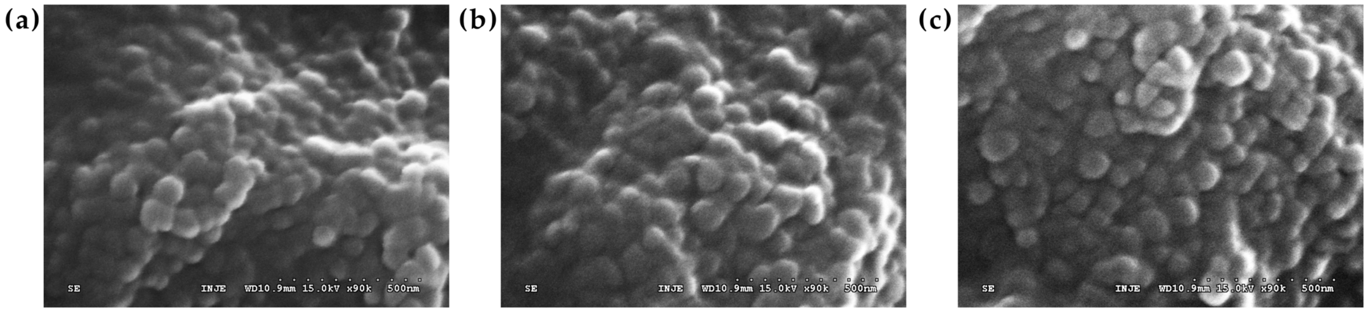

Figure S2. FE-SEM images of NPs: (**a**) F2, (**b**) F3, (**c**) F4. Scale bars represent 500nm

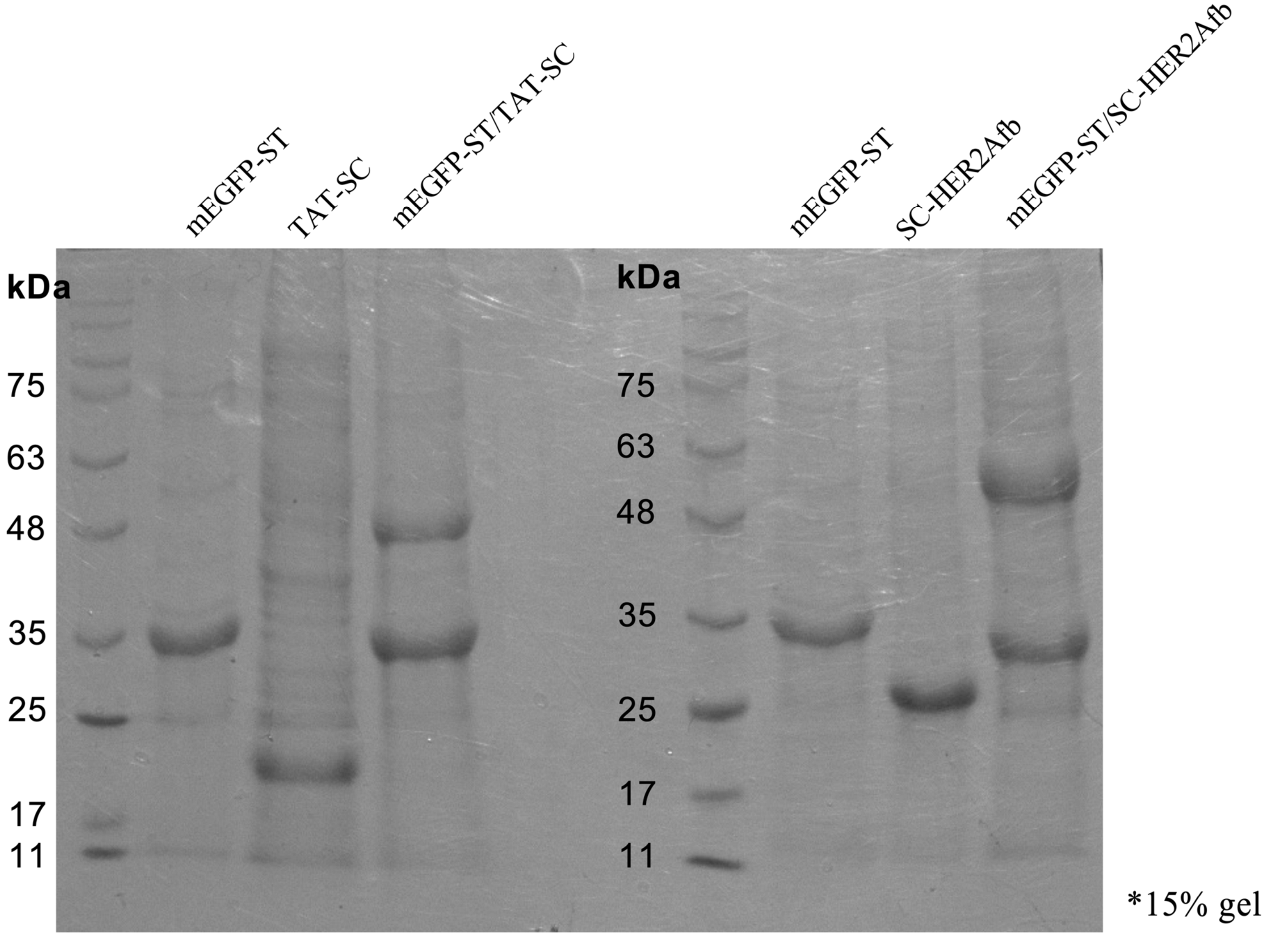

Figure S3. Sodium dodecyl-sulfate polyacrylamide gel electrophoresis (SDS-PAGE, 15%) to confirm the conjugation of mEGFP-SpyTag with TAT-SpyCatcher and HER2 affibody-SpyCatcher via the SpyTag/SpyCatcher system

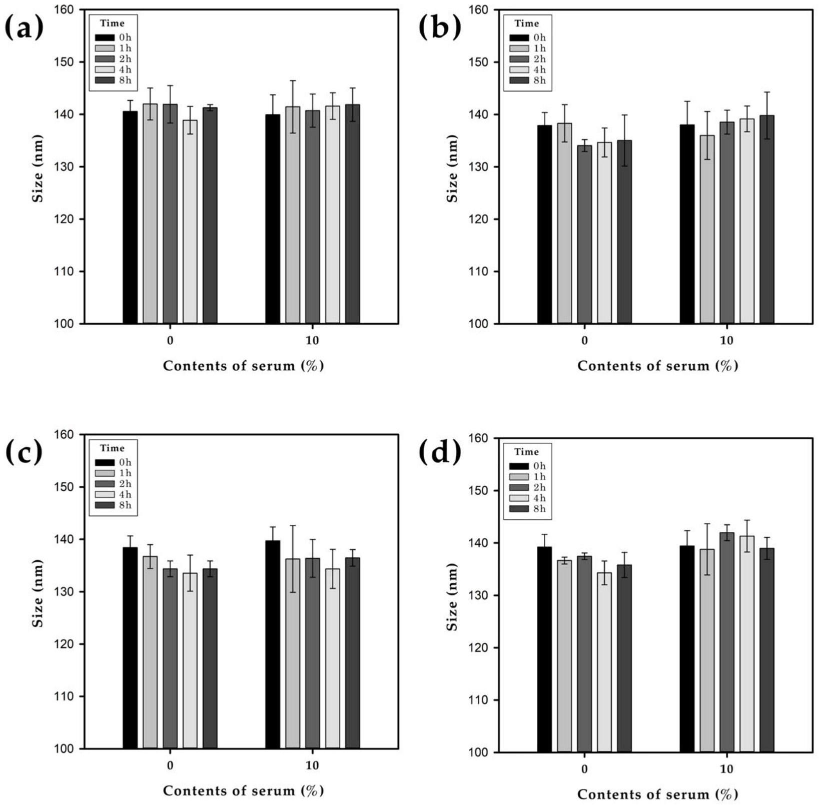

Figure S4. Serum stability of PHA NPs depending on time and serum concentration: (**a**) NP3, (**b**) NP4, (**c**) NP5 and (**d**) NP6.

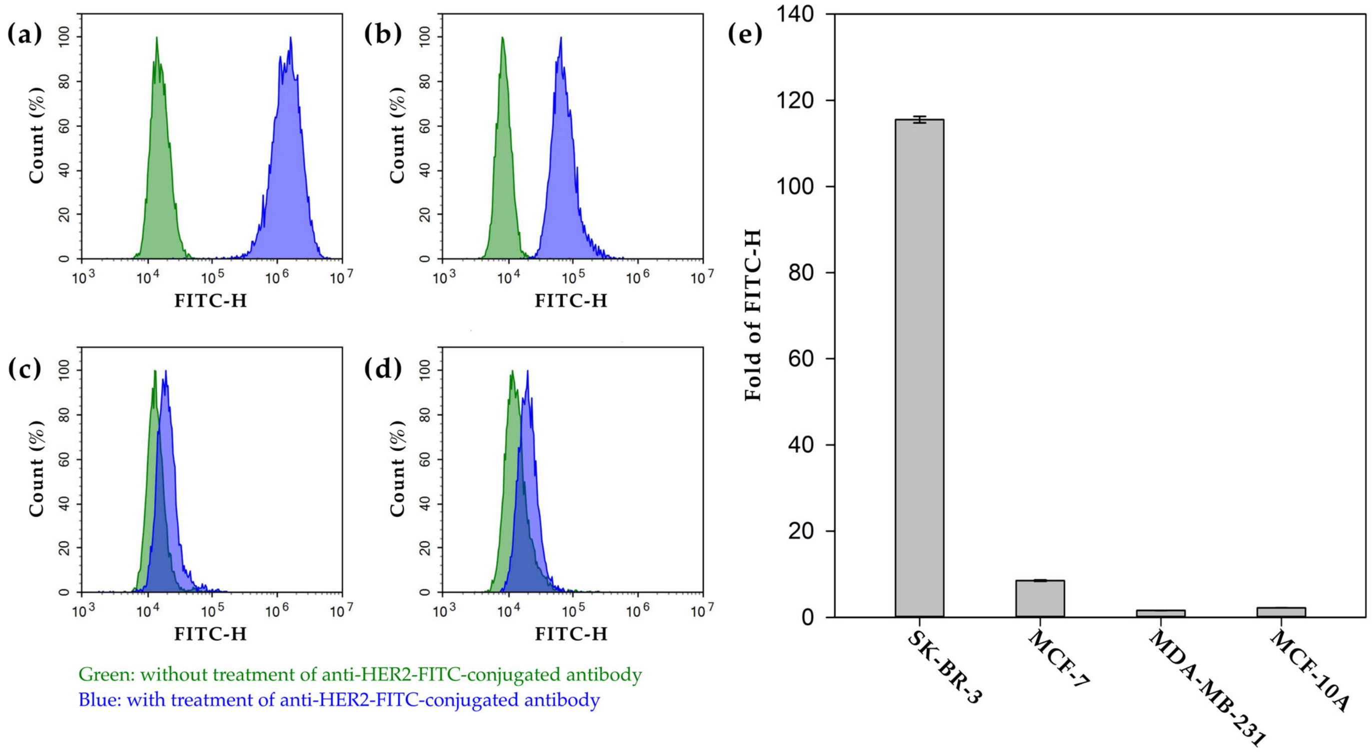

Figure S5. Flow cytometry analysis of HER2 expression levels in various cell lines: (**a–d**) FITC-H histograms of (**a**) SK-BR-3, (**b**) MCF-7, (**c**) MDA-MB-231, (**d**) MCF-10A cells and (**e**) bar graph of HER2 expression levels in each cell line using FITC-conjugated anti-HER2 monoclonal antibodies.
